# Ozone Disc Nucleolysis for Cervical Intervertebral Disc Herniation: A Systematic Review and Meta-Analysis

**DOI:** 10.7759/cureus.59855

**Published:** 2024-05-08

**Authors:** Sharad B Ghatge, Ajeya Asarkar, Sanket S Warghade, Siddhant Shirsat, Aalopa Deb

**Affiliations:** 1 Interventional Neuroradiology, Grant Government Medical College and Sir Jamshedjee Jeejeebhoy Group of Hospitals, Mumbai, IND; 2 Radiodiagnosis, Grant Government Medical College and Sir Jamshedjee Jeejeebhoy Group of Hospitals, Mumbai, IND; 3 Radiology, Grant Government Medical College and Sir Jamshedjee Jeejeebhoy Group of Hospitals, Mumbai, IND; 4 Radiology, Dr. D.Y. Patil University, Navi Mumbai, IND

**Keywords:** cervical radiculopathy, intradiscal injection, chemonucleolysis, oxygen-ozone mixture, cervical disc herniation

## Abstract

Cervical intervertebral disc herniation is a common condition and most often presents as neck or upper limb pain causing varying levels of disability and dysfunction. Percutaneous injection of ozone into the intradiscal space is a novel and minimally invasive technique for managing this condition and can be an effective alternative to surgical management. A literature search was done using the keywords ozone disc nucleolysis of cervical intervertebral lesions, and five studies were selected based on the inclusion and exclusion criteria. Meta-analysis was performed to determine safety, effectiveness, and symptomatic relief (determined based on the visual analog scale (VAS)) with the publication bias being removed.

Subjects treated with ozone therapy showed significant reduction (p < 0.0001) in VAS score as compared to baseline VAS score with a standardized mean difference of 2.78 (95% CI = 1.48 to 4.07; Z value = 4.20). Ozone nucleolysis is a minimally invasive, relatively safe, and optimally effective treatment option for reducing the pain related to cervical disc. Intradiscal ozone therapy can be considered an alternative treatment modality, and well-designed, randomized clinical trials are required to confirm the long-term superiority of ozone therapy against other treatment modalities available for cervical disc herniation.

## Introduction and background

Neck pain due to various causes leads to disability and dysfunction throughout the world [1]. Cervical disc herniation is a common reason why individuals experience discomfort in their necks. The herniation of a cervical disc happens when the nucleus pulposus moves out of position inside the intervertebral disc. This may lead to pressure on the spinal cord within the spinal canal or on nerves passing through the neural foramen. Herniated cervical discs are more prevalent in women in their 30s and 40s, and their prevalence rises with age [2]. Today, there are a variety of therapeutic options available, and they all include some degree of disc excision [3]. A little shift in volume causes a big shift in pressure, which in turn releases pressure on compressed nerve roots or spinal cord [4]. One of the minimally invasive modalities is the percutaneous injection of ozone into the intradiscal space and paravertebral muscles. The proposed mechanism of ozone therapy is the oxidation of proteoglycans present in the disc leading to the dehydration of the nucleus pulposus and in turn the reduction of intervertebral disc volume. This causes the disappearance of a herniated disc and hence relief of symptoms [5]. No other treatment approach has been shown to both reduce compression and neutralize the biochemical mechanisms that cause radiculopathy as effectively as ozone disc nucleolysis [6]. The purpose of this meta-analysis and systematic review was to determine whether or not ozone therapy for cervical disc herniation was safe and effective.

## Review

Material and methodology

**Figure 1:**
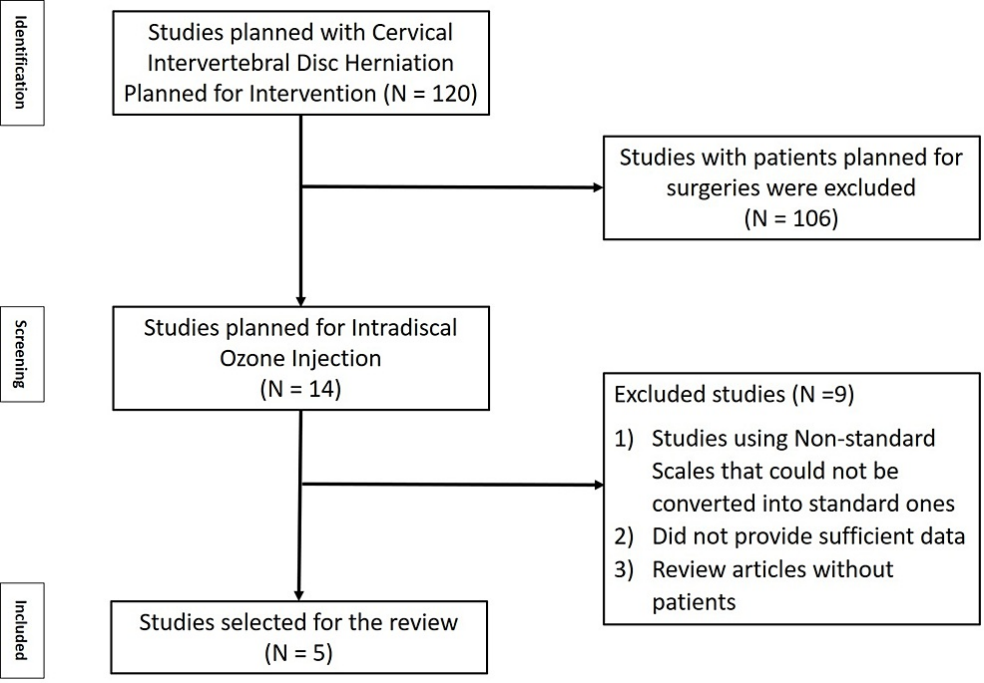
PRISMA flowchart of the study PRISMA: Preferred Reporting Items for Systematic Reviews and Meta-analysis

Following the principles laid forth by Preferred Reporting Items for Systematic Reviews and Meta-analysis (PRISMA), this study set out to examine the relevant literature and perform a meta-analysis. Literature searches were made using PubMed, ScienceDirect, Cureus, and the International Journal of Spine Surgery. Figure 1 depicts the PRISMA flowchart outlining the study selection process.

Search Strategy

The following keywords were searched to find relevant material: (a) ozone nucleolysis, (b) ozone and cervical disc, (c) ozone and herniated disc, and (d) intradiscal ozone injection. Based on these keywords, we found 26 articles in the aforementioned journals and eventually selected five articles.

Inclusion and Exclusion Criteria

We included the studies related to ozone disc nucleolysis for the cervical spine, ozone therapy for the cervical spine, and ozone oxygen mixture therapy for the cervical spine. Any procedure that did not perform intradiscal ozone injection of cervical disc herniation was excluded from the analysis. We also excluded any study that treated disc herniation in the lumbar region, those that did not have English translations, those using nonstandard scales that could not be converted to standardized scales, or those that did not provide sufficient data or could not be estimated with a statistically sound method.

Data Extraction 

The following data were extracted from the included studies and entered into a standardized table using Microsoft Excel: 1) author details, 2) sample size, 3) aims and objectives, and 4) conclusions. From each study, the baseline and six-month visual analog scale (VAS) scores were extracted.

Quality Assessment and Risk of Bias

The Joanna Briggs Institute Critical Appraisal Checklist for Quasi-Experimental Studies was used to evaluate the study's quality and the existence of bias. The tool included nine questions on the study's design; "yes" indicated high quality, "no" indicated low quality, and "unclear" meant no quality at all. Based on the quantity of "Y" chosen from the checklist, the bias risk % is computed. The formula did not take this issue into account when "NA" was chosen, following the Joanna Briggs Institute's recommendations. A very high risk of bias was indicated by less than 49% of affirmative responses. The likelihood of bias was moderate between 50% and 70% and low after 70%.

Statistical Analysis 

The meta-analyses were conducted using RevMan 5.4, which was developed by the Nordic Cochrane Centre in Copenhagen. The model used in the studies was the random effects model. A Q-test was used to evaluate heterogeneity, and I2 statistics were used to quantify it. Pre- and post-ozone treatment VAS score data (mean, SD, and sample size) were culled from relevant research. This comparison was conducted: Before and after ozone treatment, a comparison of VAS scores. A random effects model was used for analyses when the test demonstrated high heterogeneity (I2 > 50%), but a fixed effects model would be employed when I2 < 50%.

Results

We included five studies with 4432 patients, with 1256 (28.3%) patients being females and 3176 (71.7%) patients being males [6-10]. Table 1 outlines the baseline characteristics of the included studies.

**Table 1:** Study characteristics table

Study Number	Author name	Number of patients	Aims and objectives	Conclusion
1	Ghatge et al. [6]	246	Prospective study of the role of ozone disc nucleolysis in cervical intervertebral disc herniation	Ozone disc nucleolysis significantly reduced the pain related to cervical disc herniation along with a significant reduction in disability
2	Beyaz et al. [7]	44	To investigate the six-month efficacy and safety of O_2_-O_3_ mixture therapy in patients with cervical disc herniation (CDH) and chronic neck pain	Intradiscal ozone injection showed long-term favorable effects
3	Wang et al. [8]	19	This retrospective study compared the efficacy of combined percutaneous ozone injection and percutaneous discectomy to percutaneous ozone injection alone for the treatment of cervical disc herniation	Rate of effective treatment in combination more than the treatment with just ozone
4	Alexandre et al. [9]	252	To study the role of Intradiscal injection of oxygen-ozone gas mixture treatment on a series of patients affected by cervical disc pathology	This technique is simple, has no risks, and offers to the patient a solution without the discomfort of surgery and the possible risks it entails
5	Rashid et al. [10]	3871	Experience with ozonucleolysis with patients affected by pain in the cervical region (brachialgia) due to disc herniation including postoperative recurrence or disc prolapse	Intradiscal injection of ozone for herniated cervical discs has revolutionized the percutaneous approach to nerve root diseases making it safer, cheaper, and easier to repeat than treatments currently in use

Comparison of VAS Score Before and Six Months After Ozone Therapy

**Figure 2:**
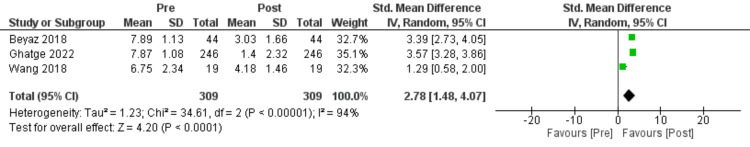
Forest plot showing the comparison of VAS score before and six months after ozone therapy p ≤ 0.05 is considered as significant

Three studies matched the criteria for quantitatively analyzing data; hence, they were included in the meta-analysis. Figure 2 is a forest plot showing the findings of the overall comparison. Heterogeneity was found to be larger than 50% (I2 = 94%), leading to the use of the random effects model in the meta-analysis of the chosen studies. By comparing their baseline VAS score to their score after ozone treatment, subjects demonstrated a substantial decrease (p < 0.0001), with a standardized mean difference of 2.78 (95% CI = 1.48 to 4.07; Z value = 4.20).

Apart from these three studies, the study by Alexandre et al. showed that pain symptomatology was completely abolished in 79.3% of the patients, and the study done by Rashid et al. showed complete recovery with the disappearance of symptoms in 60% of the patients [9,10].

Funnel Plot for the Assessment of Publication Bias

**Figure 3:**
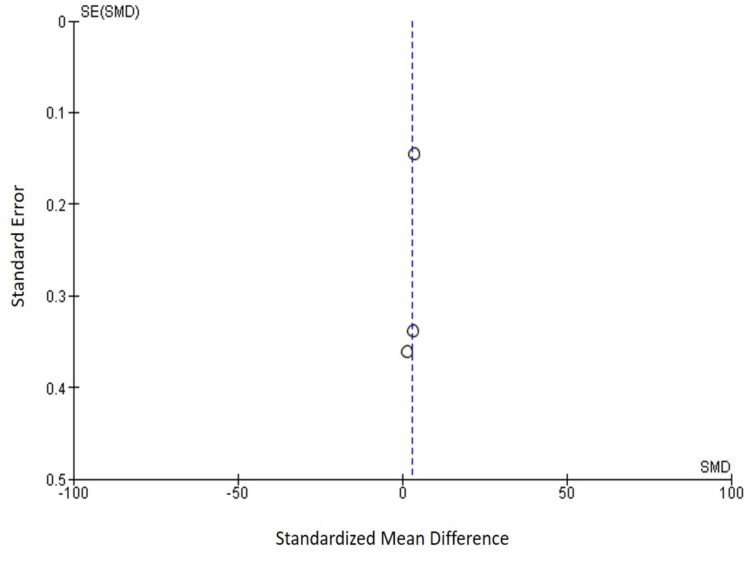
Funnel plot for the assessment of publication bias SE: Standard error; SMD: standardized mean difference

Figure 3 shows a funnel plot, which indicates a symmetric distribution of studies because all points fall within the funnel. Therefore, it may be concluded that there is no prejudice in the publication. Results of the Eggers' test also indicate the presence of funnel plot symmetry and less chance of publication bias in the study (p = 0.107).

Discussion

The meta-analysis of the studies included demonstrates the safety and effectiveness of ozone therapy for the treatment of cervical disc herniation with data from 309 patients.

The studies that were used for the analysis had inclusion criteria that encompassed a wide range of patients with ages ranging from 28 to 71 years. The efficacy of the outcome was assessed by VAS and modified Macnab grades. Scores on the VAS and modified Macnab scores were used to evaluate the results at one, three, and six months, as well as after a year.

Disc herniations happen when the nucleus pulposus pushes through the annulus fibrosus, either partially or completely. Acute arterial ischemia causes excruciating shooting pain, whereas mild compressions cause venous congestive root edema [11, 12]. The release of inflammatory mediators is caused by the nucleus pulposus, which is immune-privileged, being directly exposed to our immune system when the intervertebral disc ruptures, as it moves through a rip in the annulus fibrosus. Angiogenesis and macrophage chemotaxis are outcomes of monocyte recruitment from the immune system caused by these inflammatory mediators.

Inflammation of nerve roots and dorsal root ganglia is an undesirable consequence of lymphocyte activation, which follows the production of interferon-gamma (IFN-γ) and the recruitment of macrophages. The extruded discs include higher concentrations of inflammatory markers such as interleukin‐6 (IL‐6), IL‐12, IFN-γ, and cluster of differentiation 68 (CD68) macrophages. The immune system triggers two distinct kinds of macrophages in inflammatory responses. Prolonged and excruciating disease could result from the M1-mediated pro-inflammatory phase lingering for an extended period. Pain that travels downward along a nerve's course is known as radiating radiculopathy, and it is caused by these inflammatory cascades of events. Herniated nucleus pulposus contains substantial levels of tumor necrosis factor-α and phospholipase A2 [13,14,15]. These cause nerve roots to become more sensitive to mechanical pressure by partially demyelinating them. Hyperexcitability, brought on by mechanical compression from a herniated disc, may cause neuropathic paresthesia and discomfort. Therefore, the combination of biochemical and biomechanical variables is responsible for the symptoms that result from disc herniation [13]. Ozone is also implicated in regenerating the myelin sheath [16,17].

The degeneration and drying up of the intervertebral disc is a normal aspect of aging and may lead to chronic herniations, which usually manifest with less severe, more subtle symptoms. On the other hand, nucleus pulposus extrusion via a gap in the annulus fibrosus is often the consequence of trauma in acute herniations. Unlike chronic herniations, the symptoms of this injury often come on suddenly and are more severe.

The process of ozone nucleolysis is carried out by injecting a combination of oxygen and ozone. This combination makes use of ozone's biological capabilities. Ozone nucleolytics are thought to work by first breaking down glycosaminoglycans, which are abundant in the nucleus pulposus. This releases water molecules, which reduce the pressure inside the nucleus, which causes it to recoil and restore the intervertebral disc [6]. In addition to its anti-inflammatory effects, ozone speeds up the transition from the inflammatory to the reparative phase of macrophages in the epidural space, which is located outside of the nucleus pulposus [18]. Ozone also acts on the erythrocytes resulting in the alteration of 2,3-disphosphoglycerate (2,3-DPG) which causes a rightward shift of the hemoglobin (Hb)-O_2_ dissociation curve, meaning it decreases the O_2 _binding capacity of the Hb, and hence, more O_2_ is delivered to the tissue [19]. This decreases the ischemic damage caused to the nucleus pulposus due to venous stasis and improves the microcirculation.

No major adverse effects such as vascular or nerve damage, hematoma, or puncture infection were reported during or after the procedure in all three studies included. In Beyaz et al., one patient reported hoarseness and needed reference to a specialist; however, the symptom resolved spontaneously within a week without any intervention [7]. Ozone therapy for disc herniation is considered to be relatively safer than other modalities of treatment and has a few adverse effects at therapeutic levels of ozone therapy. The documented occurrence of adverse effects is about 0.1% [20]. According to some case reports, injuries to the eyes, headaches, and paresthesia are the most typical side effects of ozone treatment, which are transient in nature. Very rarely, problems such as pneumoencephalus, air embolism, and bilateral vitreoretinal hemorrhages may occur [21]. Possible long-term consequences of the procedure include a subcutaneous hematoma, epidural vascular puncture, and puncture trauma damage. These negative effects are not directly associated with the ozone but rather with the process of administering the ozone [6]. No cases of discitis were reported after ozone therapy, unlike all other modalities of treatment for cervical disc herniation. This is likely due to the strong oxidizing and disinfecting nature of ozone [19]. A case of cardiopulmonary arrest and vertebrobasilar stroke has been reported [22,23]. Recently reported rare but novel complications of ozone therapy include emphysema and pneumomediastinum [24].

In Beyaz et al., 44 patients (16 men and 28 women) were contacted and followed-up [7]. Favorable outcomes were obtained with patients reporting relief of symptoms at a rate of 93.1%, 95.4%, and 97.7% at the end of the second week, sixth week, and sixth month, respectively. Only one patient reported failure to achieve pain relief. The mean VAS score was 7.89 +/- 1.13 before the procedure and 2.27 +/- 1.25 at the end of the sixth month. A decrease of 73.1% in the average VAS score was observed compared to the baseline at the time of the final follow-up [7]. In Wang et al., the VAS scores were 6.75 +/- 2.34 before the procedure and 4.18 +/- 1.46 during the follow-ups [8]. According to the modified Macnab grading standards, the overall rate of effectiveness was 73.7% [8]. In Ghatge et al., the mean VAS score dropped from 7.87 pre-procedure to 3.09 after one month and 1.40 after six months [6]. Based on modified Macnab criteria, excellent recovery was seen in 56.1%, good recovery in 20.32%, and fair recovery in 8.94% of the patients. About 14.64% of the patients reported mediocre or no recovery which amounted to the failure rate [6]. The result of meta-analysis showed a significant reduction in VAS score as compared to the baseline with a standardized mean difference of 2.78 (95% CI = 1.48 to 4.07; Z value = 4.20)

## Conclusions

The results of our meta-analysis demonstrate that ozone nucleolysis is an effective therapy for cervical disc herniation that is both safe and minimally invasive. As a consequence of its inexpensive cost and high quality, it considerably lessens the discomfort associated with cervical disc herniation. When considering surgical options for individuals with cervical disc herniation, intradiscal ozone therapy should be investigated. To validate the long-term superiority of ozone therapy versus alternative treatment options for cervical disc herniation, we recommend well-designed, randomized clinical studies.

## References

[1] Safiri S, Kolahi AA, Hoy D (2020). Global, regional, and national burden of neck pain in the general population, 1990-2017: systematic analysis of the global burden of disease study 2017. BMJ.

[2] Sharrak S, Al Khalili Y (2023). Cervical Disc Herniation. Cervical disc herniation. 2023. In: StatPearls [Internet].

[3] Schoenfeld AJ, Weiner BK (2010). Treatment of lumbar disc herniation: evidence-based practice. Int J Gen Med.

[4] Andreula C, Muto M, Leonardi M (2004). Interventional spinal procedures. Eur J Radiol.

[5] Murphy K, Elias G, Steppan J (2016). Percutaneous treatment of herniated lumbar discs with ozone: Investigation of the mechanisms of action. J Vasc Interv Radiol.

[6] Ghatge SB, Shah RP, Surya N, Sankhala S, Unadkat CJ, Khan GM, Modi DB (2022). Ozone disc nucleolysis in cervical intervertebral disc herniation: a nonrandomized prospective analysis in 246 patients. J Craniovertebr Junction Spine.

[7] Beyaz SG, Sayhan H (2018). Six-month results of cervical intradiscal oxygen-ozone mixture therapy on patients with neck pain: preliminary findings. Pain Physician.

[8] Wang H, Zhou Y, Jiang Z (2018). Ozone injection with or without percutaneous microdiscectomy for treatment of cervical disc herniation. Technol Health Care.

[9] Alexandre A, Corò L, Azuelos A (2005). Intradiscal injection of oxygen-ozone gas mixture for the treatment of cervical disc herniations. Acta Neurochir Suppl.

[10] Rashid U, Rauf F, Hameedullah A, Atif M, Leonardi M (2018). Ozonucleolysis in cervical radiculopathy. Neurol Res Surg.

[11] Bowley MP, Doughty CT (2019). Entrapment neuropathies of the lower extremity. Med Clin North Am.

[12] Rhee JM, Yoon T, Riew KD (2007). Cervical radiculopathy. J Am Acad Orthop Surg.

[13] Buric J, Rigobello L, Hooper D (2014). Five and ten year follow-up on intradiscal ozone injection for disc herniation. Int J Spine Surg.

[14] Doita M, Kanatani T, Ozaki T, Matsui N, Kurosaka M, Yoshiya S (2001). Influence of macrophage infiltration of herniated disc tissue on the production of matrix metalloproteinases leading to disc resorption. Spine (Phila Pa 1976).

[15] Grönblad M, Virri J, Tolonen J (1994). A controlled immunohistochemical study of inflammatory cells in disc herniation tissue. Spine (Phila Pa 1976).

[16] Ozbay I, Ital I, Kucur C, Akcılar R, Deger A, Aktas S, Oghan F (2017). Effects of ozone therapy on facial nerve regeneration. Braz J Otorhinolaryngol.

[17] Ozturk O, Tezcan AH, Adali Y, Yıldırım CH, Aksoy O, Yagmurdur H, Bilge A (2016). Effect of ozone and methylprednisolone treatment following crush type sciatic nerve injury. Acta Cir Bras.

[18] Erario MLÁ, Croce E, Moviglia Brandolino MT, Moviglia G, Grangeat AM (2021). Ozone as modulator of resorption and inflammatory response in extruded nucleus pulposus herniation. Revising concepts. Int J Mol Sci.

[19] Ghatge S, Modi PD, Modi DB (2017). Clinical and radiological improvement following ozone disc nucleolysis: a case report. Cureus.

[20] Steppan J, Meaders T, Muto M, Murphy KJ (2010). A metaanalysis of the effectiveness and safety of ozone treatments for herniated lumbar discs. J Vasc Interv Radiol.

[21] Dall'Olio M, Princiotta C, Cirillo L (2014). Oxygen-ozone therapy for herniated lumbar disc in patients with subacute partial motor weakness due to nerve root compression. Interv Neuroradiol.

[22] Beyaz SG, Altaş C, Sayhan H (2018). Cardiopulmonary arrest and pneumoencephaly developing after epidural oxygen-ozone mixture therapy. Anesth Essays Res.

[23] Corea F, Amici S, Murgia N, Tambasco N (2004). A case of vertebrobasilar stroke during oxygen-ozone therapy. J Stroke Cerebrovasc Dis.

[24] İlhan B, Doğan H (2021). Novel complication of ozone therapy: massive emphysema and pneumomediastinum. Am J Emerg Med.

